# Supplementary material for: Effectiveness of Peer-Led Wellbeing Interventions in Retirement Living: A Systematic Review
Source: Int J Environ Res Public Health. 2021 Nov 3;18(21):11557. doi: 10.3390/ijerph182111557 (PMC8583038; doi:10.3390/ijerph182111557)
Supplement: Supplementary file 1 [file ijerph-18-11557-s001.zip › Supplementary File S1.pdf]

Supplementary File S1. Risk of bias assessments for each study.

CLUSTER RANDOMISED CONTROLLED TRIALS

Testing the Effect of Function-Focused Care in Assisted Living

Resnick et al 2011

Bias	Author's judgement	Support for judgement
Risk of bias due to randomisation process	Some Concerns	Comment: Details on the method of allocation and concealment were not provided in great depth. It is unclear how allocation was randomised, but baseline imbalances that would impact outcomes were not evident.
Risk of bias due to timing of identification or recruitment of participants	Low Risk	Comment: All participants were recruited before randomisation.
Risk of bias due to deviations from the intended interventions (effect of assignment to intervention)	Low Risk	Comment: Although residents were aware they were in a trial, it is unclear if there were aware of a different intervention arm (the attention-matched control group). Treatment conditions were separated by location and therefor had little potential to influence one-another. The authors report high intervention fidelity
Risk of bias due to deviations from the intended intervention (effect of adhering to intervention)	Low Risk	Comment: There was a balance non-protocol interventions, and no failures in implementation.
Risk of bias due to missing outcome data	Low Risk	Comment: No evidence that the results are biased is presented in this study. Intervention groups are fairly balanced at baseline, attrition is similar, and an intention-to-treat methodology was utilised to analyses outcome data.
Risk of bias in measurement of the outcome	Low Risk	Comment: All outcomes of interest were performance based.
Risk of bias in selection of reported results	Low Risk	Comment: Results reported were appropriate.
OVERALL JUDGEMENT		SOME CONCERNS

Trial feasibility and process evaluation of a motivationally-embellished group peer led walking intervention in retirement villages using the RE-AIM framework: the residents in action trial (RiAT)

Thogersen-Ntoumani et al 2019 – RiAT study

Bias	Author's judgement	Support for judgement
Risk of bias due to randomisation process	Some Concerns	Comment: Allocation was concealed until after the clusters were enrolled in the study, with random allocation practiced. However, the authors did not test for baseline differences between groups, claiming that this was not appropriate despite its wide-spread use.
Risk of bias due to timing of identification or recruitment of participants	Some Concerns	Comment: Unclear if participants identified and recruited before or after randomisation. Baseline imbalances not reported.
Risk of bias due to deviations from the intended interventions (effect of assignment to intervention)	High Risk	Comment: The groups were not randomised after all as initially planned due to insufficiently recruited number of participants.
Risk of bias due to deviations from the intended intervention (effect of adhering to intervention)	High Risk	Comment: Some of the intervention elements were not implemented continually.
Risk of bias due to missing outcome data	High Risk	Comment: Considerable level of missing data both at post-intervention and 6 month follow up. In fact, 6 month data was not analysed due to high levels of missing data. However, attrition was minimal and data was balanced between groups.
Risk of bias in measurement of the outcome	Some Concerns	Comment: Main outcomes (activity levels) were measured objectively using state-of-the-art activity monitors and unlikely to be influenced by any performance or assessor bias. Mental health and wellbeing were assessed using self-report measures only. The measurement methods were appropriate and consistent between groups. Assessors were aware of the assigned interventions.
Risk of bias in selection of reported results	Some Concerns	Comment: While data analysis was forgone regarding the 6 month follow-up data due to missing data, all measured outcomes were reported for the post-intervention data. There is therefore no evidence that the results are biased due to selectivity in reported results in this study.
OVERALL JUDGEMENT		HIGH RISK

Effects of a physical activity and nutrition program in retirement villages: a cluster randomised controlled trial

JANCEY et al 2017 – RVPANS study

Bias	Author's judgement	Support for judgement
Risk of bias due to randomisation process	Low Risk	Comment: Accessing the study's previously published protocol paper was necessary to make an informed decision about this domain. Allocation was concealed

		until clusters were enrolled in the study. Randomisation was conducted using a table of random numbers performed by an independent researcher not affiliated with the project.<br>Baseline characteristics were similar between the intervention and control participants.
Risk of bias due to timing of identification or recruitment of participants	High Risk	Comment: Participants were identified and recruited after randomisation. It also appears that eligibility criteria between participants in the intervention and control group differed. For instance, participants in the intervention group were required to complete the Physical Activity Readiness Questionnaire in order to eliminate those at high risk. It is not clear if the control were required to do the same.<br><br>It is unlikely that study staff were blinded to cluster allocation when recruiting participants.
Risk of bias due to deviations from the intended interventions (effect of assignment to intervention)	Some Concerns	Comment: Without better understanding of the participant information provided, it is unclear if participants were aware that they were in a trial, aside from knowing they were in a study. However, they were potentially aware, and study personnel were also aware of the intervention. However, there were no deviations from the experimental context and all participants completed the study in the groups to which they were assigned. The statistical analyses chosen (mixed regression) could have included all participants with at least baseline data, but seems to have only been performed on "program completers"
Risk of bias due to deviations from the intended intervention (effect of adhering to intervention)	Some Concerns	Comment: The authors do not report how many participants adhered to the intervention, and what their criteria were for "per-protocol" completers. However, analyses are based on people who successfully completed the post-test assessments.
Risk of bias due to missing outcome data	Some Concerns	Comment: Outcome data were available for all clusters, but not all participants within clusters. More participants in the intervention group were lost to follow-up due to poor health and injury than in the control group, and it is possible that intervention effects influenced this selection attrition. However, baseline characteristics of completers between the groups appear fairly similar.
Risk of bias in measurement of the outcome	Some Concerns	Comment: While the measurement method was appropriate and consistent between groups, assessors were potentially aware of the trial taking place as well as the intervention received. This could have influenced the self-reported measures, such as physical activity and diet quality. However, it is not possible for these factors to have influenced the objective measures (such as weight, blood pressure, etc) that coincide with the self-reported changes in behaviour.
Risk of bias in selection of reported results	Some Concerns	Comment: Trial was analysed mostly in accordance with the pre-specified protocol. The authors failed to report sensitivity analyses from the Intention-to-Treat model (as detailed in their protocol paper), leading to concerns that attrition between groups was not random.
OVERALL JUDGEMENT		HIGH RISK

Cluster randomized controlled trial of a multilevel physical activity intervention for older adults

KERR et al 2018 & Zlatar et al – MIPARC STUDY

Bias	Author's judgement	Support for judgement
Risk of bias due to randomisation process	Some Concerns	Comment: Allocation was concealed until the clusters were enrolled in the study. Randomisation was conducted by a statistician after a memorandum of understanding was signed and the cluster was enrolled.<br><br>Participants in the intervention group were a little younger, more were married, and walked faster compared to participants in the control group. Other characteristics were balanced between groups, but differences in other variables (including outcome measures) were not observed. There were 4 clusters assigned to the intervention, and 7 to the control group.
Risk of bias due to timing of identification or recruitment of participants	High Risk	Comment: Participants were identified and recruited after randomisation, and staff involved in the study would have been aware of cluster allocation.
Risk of bias due to deviations from the intended interventions (effect of assignment to intervention)	Low Risk	Comment: Without better understanding of the participant information provided, it is unclear if participants were aware that they were in a trial, aside from knowing they were in a study. However, they were potentially aware, and study personnel were also aware of the intervention. However, there were no deviations from the experimental context and all participants completed the study in the groups to which they were assigned. The statistical analyses chosen (intention to treat mixed regression) appropriately estimated the effect of assignment.
Risk of bias due to deviations from the intended intervention (effect of adhering to intervention)	Low Risk	Comment: Fidelity of the intervention was high, with around 80% or better (on average) attendance at group sessions, counselling calls and enactment of intervention aims.
Risk of bias due to missing outcome data	Low Risk	Comment: Outcome data were available for all clusters, there was minimal attrition, and it was balance between groups. Statistical analyses included all participants at baseline, regardless of participation status at post-test.
Risk of bias in measurement of the outcome	Low Risk	Comment: The measurement method was appropriate and consistent between groups, and although assessors were aware of the assigned interventions, the primary outcomes were all objectively assessed and unlikely to be influenced by any performance or assessor bias.
Risk of bias in selection of reported results	High Risk	Comment: The authors do not report results of a mixed model regression on all primary and secondary outcomes data with a group x time interaction. Instead, they note in text that changes in the secondary outcomes did not vary significantly over time.
OVERALL JUDGEMENT		HIGH RISK
