# Supplementary material for: Effectiveness of Peer-Led Wellbeing Interventions in Retirement Living: A Systematic Review
Source: Int J Environ Res Public Health. 2021 Nov 3;18(21):11557. doi: 10.3390/ijerph182111557 (PMC8583038; doi:10.3390/ijerph182111557)
Supplement: Supplementary file 1 [file ijerph-18-11557-s001.zip › Supplementary Table S2.pdf]

Supplementary Table S2. Reporting quality for each study based on CONSORT items.

			Study						
			Jancey et al 2017 [14]	Kerr et al 2018 [3]	Nanduri et al 2018 [28]	Resnick et al 2016 pre-post [29]	Thogersen-Ntoumani et al 2019 [31]	Zlatar et al 2019	Resnick RCT [32]
	**Title and abstract**	1a Identification as a randomised trial in the title	1	1	NA	NA	0	0	0
		1b Structured summary of trial design, methods, results, and conclusions			1	1			
**Introduction**	**Background and objectives**	2a Scientific background and explanation of rationale	1	1	1	1	1	1	1
		2b Specific objectives or hypotheses							
**Methods**	**Trial design**	3a Description of trial design (such as parallel, factorial) including allocation ratio	1	1	NA	NA	NA	1	1
		3b Important changes to methods after trial commence-ment (such as eligibility criteria), with reasons			0		1		
	**Participants**	4a Eligibility criteria for participants	1	1	1	1	1	1	1
		4b Settings and locations where the data were collected							
	**Interventions**	5 The interventions for each group with sufficient details to allow replication, including how and when they were actually administered	0	1	1	0	1	1	1
	**Outcomes**	6a Completely defined pre-specified primary and second-ary outcome measures, including how and when they were assessed	0	1	0	0	1	1	1
		6b Any changes to trial outcomes after the trial com-menced, with reasons				NA			
	**Sample size**	7a How sample size was determined	1	1	1	1	1	1	0
		7b When applicable, explanation of any interim analyses and stopping guidelines					NA		
**Randomisation:**	**Sequence generation**	8a Method used to generate the random allocation se-quence	1	1	NA	NA	NA	1	0
		8b Type of randomisation; details of any restriction (such as blocking and block size)			NA	NA	NA	1	

	**Allocation concealment mechanism**	9 Mechanism used to implement the random allocation sequence (such as sequentially numbered containers), describing any steps taken to conceal the sequence until interventions were assigned	0	1	NA	NA	NA	0	0
	**Implementation**	10 Who generated the random allocation sequence, who enrolled participants, and who assigned participants to interventions	0	1	NA	NA	NA	1	0
	**Blinding**	11a If done, who was blinded after assignment to interventions (for example, participants, care providers, those CONSORT 2010 checklist Page 2 assessing outcomes) and how	1	1	NA	NA	NA	1	0
		11b If relevant, description of the similarity of interventions			NA	NA	NA		
	**Statistical methods**	12a Statistical methods used to compare groups for primary and secondary outcomes	1	1	1	NA	1	1	1
		12b Methods for additional analyses, such as subgroup analyses and adjusted analyses				1			
**Results**	**Participant flow (a diagram is strongly recommended)**	13a For each group, the numbers of participants who were randomly assigned, received intended treatment, and were analysed for the primary outcome	1	1	NA	0	1		1
		13b For each group, losses and exclusions after randomisation, together with reasons			NA			0	
	**Recruitment**	14a Dates defining the periods of recruitment and follow-up	0	0	0	0	0	0	0
		14b Why the trial ended or was stopped							
	**Baseline data**	15 A table showing baseline demographic and clinical characteristics for each group	1	1	1	0	1	1	0
	**Numbers analysed**	16 For each group, number of participants (denominator) included in each analysis and whether the analysis was by original assigned groups	1	1	1	0	1	1	1
	**Outcomes and estimation**	17a For each primary and secondary outcome, results for each group, and the estimated effect size and its precision (such as 95% confidence interval)	1	0	1	1	1		1
		17b For binary outcomes, presentation of both absolute and relative effect sizes is recommended				NA	NA	0	

	**Ancillary analyses**	18 Results of any other analyses performed, including subgroup analyses and adjusted analyses, distinguishing pre-specified from exploratory	1	1	0	NA	1	1	0
	**Harms**	19 All important harms or unintended effects in each group	0	1	0	0	0	0	1
**Discussion**	**Limitations**	20 Trial limitations, addressing sources of potential bias, imprecision, and, if relevant, multiplicity of analyses	1	1	1	1	1	0	1
	**Generalisability**	21 Generalisability (external validity, applicability) of the trial findings	1	1	1	1	1	1	1
	**Interpretation**	22 Interpretation consistent with results, balancing benefits and harms, and considering other relevant evidence	1	1	1	1	1	1	1
**Other information**	**Registration**	23 Registration number and name of trial registry	1	1	0	0	1	1	0
	**Protocol**	24 Where the full trial protocol can be accessed, if available	1	1	1	0	0	0	0
	**Funding**	25 Sources of funding and other support (such as supply of drugs), role of funders	1	1	1	1	1	1	1
		TOTAL SCORE	19	23	14	10	17	18	14
		PERCENTAGE	76	92	67	48	68	72	56
