# Supplementary material for: Effectiveness of Peer-Led Wellbeing Interventions in Retirement Living: A Systematic Review
Source: Int J Environ Res Public Health. 2021 Nov 3;18(21):11557. doi: 10.3390/ijerph182111557 (PMC8583038; doi:10.3390/ijerph182111557)
Supplement: Supplementary file 1 [file ijerph-18-11557-s001.zip › Supplementary Table S3. Detailed extraction table.pdf]

**Supplementary Table S3.** Detailed description of included studies.

| Lead Author (year) Country *n* participants | Aim of study | Population description *% Female* *Mean (SD) age* *Inclusion/exclusion criteria* | Description of Intervention | Outcome measures | Results *Summary of findings* [type of analysis] Specific results |
|---|---|---|---|---|---|
| **Pre-Post Intervention Designs** | | | | | |
| Nanduri, Aparna (2012-2014)<br>United States<br>40 [28] | The objectives of this study were (a) to evaluate the benefits of PHB for older adults using various geriatric fitness tests that assess balance, posture, flexibility, strength, and fall risk; (b) to evaluate the benefits of the educational component of PHB by using quizzes that test knowledge on osteoporosis, nutrition, and falls prevention; and (c) to assess the feasibility of integrating PHB into a privately funded assisted living facility. | 38% were from the assisted living residence while the other 62% were community members<br><br>Assisted living<br>F= 70%<br>Age= 86.6 (5.7)<br><br>*Exclusion criteria*<br>No medical clearance from doctor<br>Score of <20 on a MMHE | Peer-leaders ran the Project Health Bones (PHB) workshops. Three 24-week classes were conducted where participants met weekly for 60 min of exercise and 20 min of education. The focus was on strength, balance, posture and flexibility. | Pre- and post-measurements<br>• Occiput-to-wall test<br>• Functional reach test<br>• 30-second chair stands<br>• Timed up and go<br>• Single leg stand<br>• Tandem stand<br>• Four-step square test<br>• Calcium intake<br>• Falls Efficacy Scale (FES)<br>• Questionnaires to measure the participants knowledge of bone health, nutrition, and falls prevention | *Participants improved their strength, balance, posture, and flexibility, resulting in a reduced risk of falls and fractures. In addition, their knowledge of bone health, nutrition, and fall prevention increased.*<br><br>[Results are presented for the Assisted Living group. P values presented as categories: *< 0.05, **<0.01, ***<0.001. Bold indicates a significant difference from baseline to mid or end point. Differences between means examined using ANOVA]<br><br>Assisted Living Geriatric Assessment results<br>Test: Baseline score ± SD – Midpoint score ± SD – 24-week score ± SD<br>Functional reach: 7.7 ± 3.9 – 9.8 ± 3.6 – 10.7 ± 5.2<br>Occiput to wall: 6.2 ± 3.4 – 5.5 ± 3.6 – 4.7 ± 3.0<br>**Timed up and go:** 16.5 ± 7.4 – **14.8 ± 6.1*** – 14.9 ± 8.6<br>**Four-step square:** 18.8 ± 5.5 – **15.2 ± 4.8**** – 13.7 ± 5.2<br>30s chair stand: 8.0 ± 5.0 – 8.0 ± 5.0 – 10 ± 6<br>**Tandem stand:** 15.5 ± 18.4 – **34.5 ± 38.3*** – 31.9 ± 46.3<br>Single-leg stand, eyes open: 4.5 ± 5.7 – 7.2 ± 7.6 – 6.8 ± 6.6<br><br>Assisted living questionnaire results and fitness results<br><br>Variable: Baseline score (SD) – 24-week score (SD) |

| | | | | | |
|---|---|---|---|---|---|
| | | | | | Total body weight: 147+-28(n=17) : 147+-30(n=15) : 0<br>Falls efficacy scale score: 30+-8(n=17) : 30+-9(n=10) : 0<br>Falls concern scale score: 4.7+-2.6(n=17) : 3.5+-2.3(n=15) : 1.2<br>**Percent correct quiz scores: 72.1+-9.2(n=16) : 84.4+-9.1(n=15) : 12.3*****<br>Calcium intake>goal: 2063(n=1) : 1636(n=1) : 427<br>Calcium intake<goal: 715+-198(n=5) : 726+-119(n=5) : 11<br>**Weight lifted by participants (Arm): 0(n=17) : 2.7+-1.4(n=15) : 2.7*****<br>**Weight lifted by participants (Leg): 0(n=17) : 2.4+-1.2(n=15) : 2.4***** |
| Resnick, Barbara (2016) United States 3676 [29] | The purpose of this study was to disseminate and implement the previously established, effective FFC-AL approach to 100 AL settings. | The size of the facilities ranged from 11 to 265 beds, with an average size of 49 (SD = 47) beds.<br>F = not reported<br>Age = not reported<br><br>*Inclusion criteria*<br>Settings were in Maryland and within 150miles of Baltimore<br>Willing to identify a nurse to participate<br><br>*Exclusion criteria*<br><8 beds | The intervention involves teaching the function-focused care champions in each of the participating settings to implement the following four components of function-focused care: (1) Environment and Policy Assessments; (2) Education of staff, residents, and families, including use of our Function Focused Care website, which has six short video coaching sessions; (3) Developing Function-Focused Service Plans for Residents; and (4) Mentoring and Motivating | Primary outcome measures of interest followed the RE-AIM model and included:<br>• Reach: recruitment of settings, number of residents<br>• Effectiveness: evidence of change in the environment, policy etc<br>• Adoption: willingness to adopt intervention<br>• Implementaiton: fidelity of the intervention<br>• Maintenance: Adherence to the intervention at 12months<br><br>Results also included changes in number of falls, hospitalisations and emergency room presentations | *The intervention was marginally successful in reducing the number of falls experienced by participations, from 12 falls in a 2 month period prior to the intervention, down to 9.33 in a 2 month period after the intervention. No other health outcome variables were impacted by the intervention.*<br><br>[Chi-squared and single group ANOVA was used to compare means in outcomes before and after the intervention]<br><br>Variable: Baseline (SD) - follow-up (SD); F-test (p value)<br>**Falls: 12.00 (16.21) - 9.33 (16.40); 4.1 (0.05)**<br>Hospitalizations: 2.60 (2.61) - 2.27 (4.76); 0.11 (0.74)<br>Emergency room visits: 1.69 (1.25) - 1.92 (2.43); 0.09 (0.76)<br>**Policy: 4.15 (3.70) - 10.79 (1.67); 78.22 (0.001)**<br>**Environment: positive subscale: 9.17 (1.71) - 10.97 (1.62); 22.34 (0.001)**<br>Environment: negative subscale: 0.12 (0.33); 0.03 (0.17) - 3.19 (0.08) |
| **Cluster Randomised Controlled Trials** | | | | | |

| | | | | | |
|---|---|---|---|---|---|
| Resnick, Barbara (2011)<br>Unites States<br>171 [32] | To develop and test the Function-Focused Care in Assisted Living (FFC-AL) intervention so as to alter the decline that older adults in AL experience. | 80% Female<br>87.7 years (+-5.7 years)<br><br>*Inclusion criteria*<br>Residents were eligible to participate if they were aged 65 and older, currently living in the AL community, not in hospice or rehabilitation, and scored at least 11 on the Mini-Mental State Examination (MMSE)<br><br>*Exclusion criteria*<br><br>Life expectancy < 6 months | As above<br><br>*Control group:*<br><br>Attention matched education intervention | Outcome measures (assessed at baseline, 4 and 12 months):<br>• Physical activity (ActiGraph over 24 hours)<br>○ Time in moderate intensity activity<br>○ Kilocalories over 24 hrs<br>• Function (Barthel index and Tinetti scale)<br>• Residents beliefs about function and physical activity)<br>○ Self-efficacy for Functional Activity scale<br>○ Outcome Expectations for Functional Activity scale<br>○ Self-efficacy for Exercise scale<br>○ Outcome Expectations for Exercise scale<br>• Depression (5-item Geriatric Depression Scale)<br>• Resillience (14-item Resillience scale) | *Differences in outcomes between the control and intervention groups in this study were minimal. The only statistically significant difference was that the intervention group had better function at 12 months (determined by the Barthel Index) compared to baseline.*<br><br>[Generalized estimating equations were used to perform repeated-measures analyses, with outcome measures as the dependent variable. An intention-to-treat paradigm was followed]<br><br>Variable (time): Control Mean (Standard Error) – intervention Mean (Standard Error); p-value for difference<br><br>Moderate intensity physical activity<br>Baseline: 0.51 (0.18) – 0.43 (0.14); p = 0.71<br>4 months: 0.36 (0.13) – 1.00 (0.36); p = 0.08<br>12 months: 0.42 (0.16) – 0.72 (0.33); 0.41<br><br>Activity Counts:<br>Baseline: 36,982 (3,731) – 39,514 (4,213); p = 0.65<br>4 months: 36,834 (4,778) – 40,668 (5,859); p = 0.61<br>12 months: 32,563 (4,139) – 46,960 (6,834); p = 0.07<br><br>Physical Function (Barthel Index)<br>Baseline: 71.37 (1.23) – 73,52 (1.01); p = 0.18<br>4 months: 76.32 (0.85) – 75.48 (0.88); p = 0.49<br>**12 months: 64.42 (1.69) – 69.19 (0.35); p = 0.01**<br><br>Physical Function (Walking 50 yards)<br>Baseline: 13.20 (0.54) – 12.39 (0.58); p = 0.31<br>4 months: 12.78 (0.74) – 13.62 (0.45); p = 0.33<br>12 months: 12.59 (0.79) – 13.72 (0.50); p = 0.23<br><br>(Depression, resilience and belief scale results not reported) |

| | | | | | |
|---|---|---|---|---|---|
| ThÃgersen-Ntoumani, Cecilie (2018) Australia 116 [31] | The aims of the present study were to (1) examine trial feasibility and acceptability of the RiAT intervention, and (2) evaluate the processes involved in the implementation of the RiAT intervention using the RE-AIM framework. | F= 92%<br>Age = 78.37 (8.3)<br><br>Recruited from14 retirement villages<br><br>*Inclusion criteria*<br>Age is 60+<br>Permeant village resident<br>English speaking<br>Ability to consent<br>No terminal illness or health problems<br>Able to walk for 15mins continuously<br><2 falls in 3months<br>Does not currently meet Australian Government physical activity guidelines.<br><br>Recruited through posters, word of mouth, information sessions and so on, within the villages | 16-week cluster randomised controlled design. Physically active residents were trained to lead group walks in their villages using 10 supportive themes. Walkers attended a workshop, received a folder providing basic information about current physical activity recommendations, benefits of walking, a logbook and motivation skills training.<br><br>*Control group:* Peer-led walking groups as in the intervention arm, but peer-leaders not trained in motivational techniques | Primary outcome measures of interest followed the RE-AIM model and included (see above)<br>Efficacy outcomes included:<br>Physical activity:<br>• activPAL Micro 3 device (baseline, 16 weeks, 6 months). Examined:<br>○ Sedentary time<br>○ Step counts<br>○ Stepping time<br>○ Light physical activity<br>○ Mod-vig physical activity<br>○ Standing time<br>Mental health and wellbeing<br>• General health; SF-12 (further divided in physical health component and mental health component scores)<br>• Quality of life; Dartmouth CO-OP charts<br>• Hospital Anxiety and Depression Scale<br>• Lonliness scale<br>• Subjective vitality scale | *Walkers in the main experimental condition marginally increased their step count, although there were no group differences in other measures, including mental health and wellbeing outcomes.*<br><br>[Results represent changes in outcomes from baseline to post assessment for the main experimental group (Ambassador + modivational interview) using linear mixed modelling adjusted for village clustering. Between group differences were not reported]<br><br>Variable: Baseline coefficient (SE) – Post coefficient (SE); p-value<br><br>PCS: 40.14 (3.61) – 38.49 (1.98); p=0.41<br>MCS: 50.16 (2.99) – 50.36 (2.20); p=0.62<br>Physical fitness: 3.57 (0.28) – 3.28 (0.27); p=0.28<br>Emotional functioning: 4.14 (0.22) – 4.21 (0.21); p=0.75<br>Daily activities: 3.91 (0.22) – 3.99 (0.22); p=0.72<br>Social role functioning: 4.12 (0.19) – 4.21 (0.21); p=0.67<br>Perceived pain: 3.65 (0.26) – 3.52 (0.24); p=-.60<br>Change in health: 3.67 (0.23) – 3.48 (0.22); p=0.40<br>Overall health: 3.72 (0.21) – 3.81 (0.24); p=0.72<br>Social support: 4.23 (0.27) – 4.15 (0.3); p=0.79<br>Quality of life: 4.33 (0.17) – 4.27 (0.17); p=0.73<br>Anxiety: 1.75 (0.15) – 1.7 (0.14); p=0.74<br>Depression: 1.67 (0.14) – 1.69 (0.13); p=0.86<br>Loneliness: 2.51 (0.17) – 2.43 (0.14); p=0.59<br>Subjective vitality: 4.20 (0.29) – 4.43 (0.32); p=0.46<br>**Steps (per day): 7088 (649) – 8077 (461); p=0.05**<br>**Stepping time: 94 (8) – 106 (5); p=0.04**<br>LIPA: 317 (28) – 329 (14); p= 0.41<br>MVPA: 56 (6) – 65 (4); p=0.07<br>Sitting: 540 (29) – 537 (23); p=0.89<br>Standing: 280 (25) – 292 (13); p=0.36 |
| Jancey, Jonine (2017) Australia | To determine if a 6- month home-based intervention could improve the physical | F=74.6%<br><br>Age = 72 (5.2) | A six-month intervention program. The program incorporated a number of | Collected at baseline and post assessment: | *The intervention group demonstrated significant increases in moderate-intensity physical activity, engagement in strength exercises, frequency of fruit consumed as well as* |

| | | | | | |
|---|---|---|---|---|---|
| 363 [14] | activity and dietary behaviours of adults aged 60 to 80 years living in retirement villages located in Perth, Western Australia | Recruited from 38 retirement villages<br><br>*Inclusion criteria*<br>No special diet<br><150min mod-intensity PA per week<br>Not participating in other physical activity programs<br>Retirement villages had to have residents aged 60-80 and >50 beds.<br><br>Participants were recruited through village information sessions and written information material. | educational resources (booklet, calendar, exercise chart, resistance bands and bi-monthly newsletters) based on the National Physical Activity and Dietary Guidelines, and trained program ambassadors of a similar age. The program ambassadors were responsible for two face-to-face meetings whereby they introduced the program, and distributed and explained the program resources. The face-to-face introduction discussed goal setting, included a demonstration of the exercise program and responded to any questions. This was then followed-up by regular telephone contact by the program ambassadors.. Motivational interviewing by these ambassadors via telephone contact supported participant goal setting, adherence and program sustainability.<br><br>*Control group:*<br>No intervention | • International Physical Activity Questionnaire (IPAQ):<br>○ Walking<br>○ MVPA<br>○ Vigorous activity<br>○ Strength exercise (y/n)<br>○ Sitting time<br>• Fat and Fibre Barometer (FFB)<br>○ Fruit intake<br>○ Vegetable intake<br>○ Fibre intake<br>○ Fat intake<br>○ Fat avoidance score<br>• Height<br>• Weight<br>• Waist and hip circumference<br>• BMI<br>• Blood pressure | *fat avoidance and fibre intake scores, in addition to a 0.5 kg mean reduction in weight post program, whereas no apparent changes were observed in the control group. Mixed regression results further confirmed statistically significant improvements in weight loss (p < 0.05), engagement in strength exercises (p < 0.001) and fruit intake (p = 0.012) by the intervention participants at post-test relative to their controls.*<br><br>[Mixed regression analysis: group x time<br>Variable: Regression coefficient (standard error), p-value]<br><br>**Weight: -0.53 (0.27), p=0.047**<br>BMI: -0.03 (0.17), p=0.855<br>Waist to hip ratio: -0.01 (0.01), p=0.243<br>Systolic blood pressure: -0.79 (2.41), p=0.743<br>**Diastolic blood pressure: -2.16 (1.01), p=0.032**<br>Walking time: 0.23 (0.2), p=0.257 (linear mixed regression and logarithmic transformed)<br>**Sitting time: -0.11 (0.05), p=0.027** (gamma mixed regression model)<br>Moderate activity: 0.07 (0.21), p=0.726 (gamma mixed regression model)<br>Vigorous activity: -0.26 (0.40), p=0.513 (logistic mixed regression model)<br>**Strength exercise: 1.14 (0.31), p=<0.001** (logistic mixed regression model)<br>**Frequent fruit intake: 0.63 (0.25), p=0.012** (logistic mixed regression model)<br>Frequent vegetable intake: 0.39 (0.45), p=0.378 (logistic mixed regression model)<br>Fibre intake score: 0.07 (0.05), p=0.189<br>Fat intake score: 0.04 (0.04), p=0.285<br>Fat avoidance score: -0.06 (0.07), p=0.418 |
| Kerr, Jacqueline (2010-2014) United States 307 [3] | To determine if interventions in residential settings | Control<br>F= 71%<br>Age= 83.4 (6.5) | Activities included up to 9 group education sessions (interpersonal level) over a 6-month period at sites. | Completed at baseline, 3,6,9,12 months for 6 days<br><br>Primary outcomes | *PA significantly increased in the intervention condition compared with the control condition and remained significantly higher across the 12 month study. Men and partici-* |

| | | | | | |
|---|---|---|---|---|---|
| | support a multi-level approach to behaviour change. | Intervention<br>F= 74%<br>Age= 81.5 (5.9)<br><br>Recruited from 11 retirement villages<br><br>*Inclusion criteria*<br>Aged 65+<br>Can complete timed up and go test in <30s<br>Able to walk 20m without assistance<br>No falls in 12mnths<br>No plans to move<br>Can talk over phone<br>Retirement villages needed >100 residents and have a store or park within 1mile | Group sessions aimed to provide information, social support, and behaviour modelling. Group walks were co-led by UCSD staff and peer leaders for the first 6 months, with the peer leaders continuing this after 6 months. Four counselling phone calls were completed in the first 8 weeks to identify barriers and support safe goal setting (individual level). Participants wore pedometers and completed weekly step logs.<br><br>*Control group:* | • Physical activity (GT3X accelerometer)<br><br>Completed at baseline, 6 and 12 months<br><br>Objective secondary outcomes:<br>• Short physical performance battery (SPPB)<br>• Timed 400 m walk test<br>• Blood pressure<br><br>Exploratory self-reported outcomes:<br>• Depression (CES-D)<br>• Perceived quality of life scale<br>• Perceived stress scale<br>• Fear efficacy scale<br>• PROMIS pain interference scale<br>• Late life function and disability instrument | *pants under 84 years old benefited most from the intervention. There was a significant decrease in systolic and diastolic blood pressure at 6 months. Physical functioning improved but the changes were not statistically significant.*<br><br>Figure 2 shows there is a statistically significant difference in MVPA between intervention and control groups as there is no overlap of confidence intervals between the two conditions.<br><br>We need to contact the authors in order to obtain all statistics on the outcomes of interest |
| Zlatar, Zvinka (2019)<br>United States<br>307 [30] | The authors investigated if the physical activity increases observed in the Multilevel Intervention for Physical Activity in Retirement Communities (MIPARC) improved cognitive functions in older adults. The authors also examined if within-person changes in moderate to | Control<br>F= 71%<br>Age= 85.33 (6.6)<br><br>Intervention<br>F= 74%<br>Age= 81.9 (5.9)<br><br>*Inclusion criteria*<br>Age 65+ | Interventions were led by study staff for the first 6 months and sustained by peer leaders for the next 6 months. Components included individual counselling and self-monitoring with pedometers, group education sessions, and printed materials. | Completed at baseline, 3,6,9,12 mnths for 6 days<br><br>Primary outcomes<br>• Physical activity (GT3X accelerometer)<br><br>Completed at baseline, 6 and 12 months | *There were no significant differences in cognitive functions between the intervention and control groups at 6 or 12 months. However, within person increases in MVPA, and not low- light or highlight PA, were associated with improvements in performance in the entire sample.*<br><br>Intervention effects on cognition (condition x time interaction)<br>6 Months<br>Variable: B(SE), p |

| | | | | | |
|---|---|---|---|---|---|
| | vigorous physical activity (MVPA), as opposed to low-light and high-light physical activity, were related to cognitive improvements in the entire sample | Can complete timed up and go test in <30s<br>Able to walk 20m without assistance<br>Can talk over phone<br>Obtained doctor permission to participate<br>Accurately complete a post-consent comprehension test<br><br>*Exclusion criteria*<br>Unable to comply to device wearing protocols<br>Plans to move within a year<br>Fall in the past 12mnths | | • Trail Making Test (TMT) Parts A and B (assessment of cognitive flexibility/executive function<br><br>• The Wechsler Adult Intelligence Scale IV Symbol Search Test (assessment of psychomotor speed/visual scanning | TMT Part A: -0.09(0.06), p=0.15<br>TMT Part B: 0.00(0.07), p=0.97<br>TMT Part A-B: 0.03(0.11), p=0.76<br>Symbol Search: -0.75(1.25), p=0.55<br><br>12 Months<br>TMT Part A: -0.09(0.07), p=0.17<br>TMT Part B: 0.02(0.08), p=0.76<br>TMT Part A-B: 0.10(0.11), p=0.39<br>Symbol Search: -0.53(1.30), p=0.69<br><br>All data was log-transformed. Need to add within person changes |
